# Osteopontin Levels in Human Milk Are Related to Maternal Nutrition and Infant Health and Growth

**DOI:** 10.3390/nu13082670

**Published:** 2021-07-31

**Authors:** Aysegül Aksan, Izzet Erdal, Siddika Songül Yalcin, Jürgen Stein, Gülhan Samur

**Affiliations:** 1 Institute of Nutritional Science, Justus-Liebig University, 35392 Giessen, Germany; ayseguel.aksan@ernaehrung.uni-giessen.de; 2 Department of Nutrition and Dietetics, Faculty of Health Sciences, Hacettepe University, Sihhiye, Ankara 06100, Turkey; gsamur@hacettepe.edu.tr; 3 Institute of Pharmaceutical Chemistry, Goethe University, 60438 Frankfurt am Main, Germany; 4 Department of Pediatrics, Faculty of Medicine, Hacettepe University, Altindağ, Ankara 06230, Turkey; izzet.erdal@gmail.com (I.E.); siyalcin@hacettepe.edu.tr (S.S.Y.)

**Keywords:** osteopontin, breast milk, human milk, mature milk, maternal diet, infant health, immune system

## Abstract

*Background:* Osteopontin (OPN) is a glycosylated phosphoprotein found in human tissues and body fluids. OPN in breast milk is thought to play a major role in growth and immune system development in early infancy. Here, we investigated maternal factors that may affect concentrations of OPN in breast milk, and the possible associated consequences for the health of neonates. *Methods:* General characteristics, health status, dietary patterns, and anthropometric measurements of 85 mothers and their babies were recorded antenatally and during postnatal follow-up. *Results:* The mean concentration of OPN in breast milk was 137.1 ± 56.8 mg/L. Maternal factors including smoking, BMI, birth route, pregnancy weight gain, and energy intake during lactation were associated with OPN levels (*p* < 0.05). Significant correlations were determined between body weight, length, and head circumference, respectively, and OPN levels after one (r = 0.442, *p* = < 0.001; r = −0.284, *p* = < 0.001; r = −0.392, *p* = < 0.001) and three months (r = 0.501, *p* = < 0.001; r = −0.450, *p* = < 0.001; r = −0.498, *p* = < 0.001) of lactation. A negative relation between fever-related infant hospitalizations from 0–3 months and breast milk OPN levels (r = −0.599, *p* < 0.001) was identified. *Conclusions:* OPN concentrations in breast milk differ depending on maternal factors, and these differences can affect the growth and immune system functions of infants. OPN supplementation in infant formula feed may have benefits and should be further investigated.

## 1. Introduction

Breast milk is a unique source of nutrients that is physiologically tailored to meet the changing needs of the infant during the first six or more months of life [1,2,3,4]. In addition to providing optimal energy and nutrition, breast milk optimally manages the transition of the neonate to extrauterine life through a combination of bioactive proteins, lipids, oligosaccharides, and immunomodulatory components [5,6].

Epidemiological studies have shown that breastfed infants are less likely to develop necrotizing enterocolitis, leukemia, and lymphomas, infectious diseases and allergies, or immune-mediated diseases such as asthma, celiac disease, or diabetes, than infants unable to be breastfed for a variety of reasons [6,7,8,9]. Furthermore, the intestinal microbiota of breastfed infants has been shown to differ from that of non-breastfed babies. Microbial dysbiosis in early life has been suggested to correlate with an increased incidence of immune-modulated disease such as asthma and atopic disease, obesity, and neurodevelopmental disorders [7,8,9,10]. Infants fed breast milk have also been shown to have advantages with regard to cognitive development [11].

The superiority of human breast milk over animal milks or infant formulas is thought to be due to its higher concentration of bioactive ingredients [3,5,12]. Antibacterial and opioid agonist effects of peptide components of breast milk such as lactoferrin, lactoperoxidase, lysozyme, IgA, α-lactalbumin and casein, as well as immunostimulatory effects, have been described [13,14,15,16]. However, osteopontin (OPN), a potential bioactive component, has received less attention to date, and its biological functions in breast milk have yet to be fully elucidated [17].

OPN is a glycosylated phosphoprotein that can be synthesized in many different tissues and is also found in body fluids such as urine, blood, and milk [18,19,20]. As OPN undergoes various types of post-translational modification, alternative translation and proteolytic separation specific to different tissues, organs, and body fluids, it can acquire a site-specific function. Breast milk, cord blood, and infant plasma contain exceptionally large amounts of OPN, suggesting that it may play an important role in lactogenesis and/or in immune and nervous system development and the programming of functions vital to long-term health of the neonate [13]. However, the mechanisms of direct and/or indirect involvement of OPN in these functions, are not fully understood [17]. In vitro studies have shown that breast milk OPN is partially resistant to proteolysis in the infant intestinal tract, suggesting that OPN is potentially a bioactive component [21].

Donovan et al. [22] observed that in the first trimester of life, gene expression in infant Rhesus monkeys receiving formula feed with adjuvant OPN was similar to that of breastfed infant monkeys. OPN is thought to be associated with cell cycle programming (e.g., cut homeobox gene 1 (CUX1)), intercellular communication, cell mobility, cell survival (e.g., epidermal growth factor receptor (EGFR)), and digestive system regulation (forkhead box (FOX) genes). The ability of dietary OPN to bind to integrin proteins and its well-defined association with CD44 also reinforce the view that OPN affects many related genes and pathways [22].

OPN is highly expressed throughout the lactation period: its levels were recently shown by Goonatilleke et al. to be at their highest in the second week postpartum, gradually decreasing to approximately 50% of this concentration (similar to colostrum levels) by the 24th week of lactation [23]. While the number of macrophages in breast milk is also known to decrease during lactation, it remains unclear whether it is the macrophages or the epithelial cells of breast tissue that produce the majority of breast milk OPN. However, there is evidence that the presence of OPN-producing epithelial cells in breast milk during active lactation influences immune system development in neonates [17].

The question of whether OPN supplementation in infant formula may be beneficial has recently begun to be addressed. To date, there are limited but important data to support this idea. In one controlled, double-blinded study by Lönnerdal et al. [24], 240 infants were fed for the first 6 months of life with either a whey-based standard formula or the same compound supplemented with 65 mg/L or 130 mg/L bovine OPN, or with human breast milk. At four months, infants fed standard formula had higher serum TNF-α and interleukin-2 concentrations compared with infants fed OPN-enhanced formula or breast milk. In support of this finding, infants fed OPN-supplemented formula had less frequent occurrence of febrile illness during the first 6 months of life than infants fed the standard formula. Neither the incidence of fever nor the levels of inflammatory markers were found to differ significantly between breastfed babies and those receiving OPN-supplemented formula. High TNF-α levels found in infants fed standard formula indicate a proinflammatory response to early formula use [25]. In contrast, babies receiving OPN-supplemented formula showed TNF-α levels similar to those of breastfed infants, further supporting a positive role of OPN in immune system development.

In summary, recent research has demonstrated that OPN has different tissue-specific functions, and that its levels are high in breast milk. Since breast milk OPN is presumed to be associated with immune system development, other possible biological functions of breast milk are increasingly under investigation [17,26]. However, studies that have examined the structure and function of OPN in breast milk are scarce, and few comprehensive studies have determined OPN concentrations in breast milk [13]. To determine whether OPN should be added to infant formulas, however, it is important to determine natural OPN levels in human breast milk samples.

While the composition of breast milk is largely determined by maternal factors including age and duration of lactation, it is additionally influenced by maternal nutrition. This study aimed to determine OPN levels in mature breast milk, to investigate maternal factors which may influence OPN levels, and to identify possible relationships between breast milk OPN levels and neonatal health. The secondary objectives were to investigate possible relationships of breast milk OPN concentrations with maternal dietary patterns and the incidence of infection in neonates.

## 2. Materials and Methods

### 2.1. Study Population

This study was conducted between June 2018 and January 2019. All mothers aged 19–40 years who applied to the Social Pediatrics Unit at the Hacettepe University Faculty of Medicine, Ankara, Turkey, between the specified dates for postnatal follow-up were invited to participate. Of these, 88 mothers who gave written informed consent were included in the study. Since the majority (85/88) of mothers were in the third month of lactation, one mother in the second month of lactation and two in the fourth month of lactation were excluded for reasons of conformity. Therefore, the study population consisted of 85 mothers in the third month of lactation. The exclusion criteria were: (1) multiple birth, (2) pregnancy complications (e.g., gestational diabetes, preeclampsia, gestational hypertension, or abnormalities concerning the birth weight or subsequent anthropometric measurements of the baby), (3) chronic illness, (4) physical or mental disability, (4) discontinuation of breastfeeding and (5) irregular attendance at, or interruption of, the follow-up visits and routine tests at the Social Pediatrics Unit. The research protocol was evaluated by Hacettepe University Ethics Committee for Non-Interventional Studies and approved in June 2018 with the approval number GO18/568-29.

### 2.2. Study Protocol

Women attending Hacettepe University’s Social Pediatric Department who were actively breastfeeding and potentially suitable study subjects according to the given inclusion/exclusion criteria were personally informed in detail about all aspects of the study, including its purpose, the planned study procedures, and personal data protection. Individuals who agreed to participate in the study were asked to read and sign the informed voluntary consent form. Those included in the study were given a morning appointment for an initial interview and sample collection at the Social Pediatrics Unit, timed to coincide with the second feed of the day.

Before the interview, previous medical records of the participants from the hospital were retrieved and information on the overall health of mother and baby was documented in a research file created for this purpose. The following data were documented: initial weight of the mother at pregnancy, weight gain during pregnancy, health conditions during pregnancy, the child’s body weight (BW), body length, and reports from postnatal examinations, vaccination programs, growth monitoring, diseases, and symptoms.

During the interview, the participant was questioned regarding any events or conditions whose documentation was incomplete or inconsistent in the prepared file, so that the records could be completed or clarified. Then, by means of a standardized questionnaire, sociodemographic characteristics, pre-pregnancy weight, weight gain during pregnancy, and health problems during pregnancy and lactation (anemia, infections, etc.) were recorded. In addition, the height and BW of the mothers and babies, and the head circumference of the babies, were measured during this interview and recorded in the questionnaire form. Pre-pregnancy body mass indices (BMI) of the mothers were calculated and categorized using the reference values of the World Health Organization (WHO) [27]. Weight gain during pregnancy was calculated and assessed according to the recommendations of the US Institute of Medicine (IOM) for healthy weight gain during pregnancy (Table 1) [28].

Nutritional intake over the past 3 months (lactation period) was assessed by means of the Food Frequency Questionnaire (FFQ) validated in 2015 by Gunes et al. to assess dietary intake in Turkish adults [29] and double-checked by 24 h-recall. In 98% of cases, 24 h-recall of food intake was in line with the FFQ. In the other 2%, the participants were questioned further, and the data was modified accordingly.

After completion and clarification of the records and completion of the questionnaire and anthropometric measurements, samples were collected; as mentioned above, all appointments were planned according to the mother’s individual daily schedule to temporally coincide with the second feed of the day. The participants were asked to breastfeed their babies from one breast (average duration: 10 min) so that both mother and baby were relaxed, after which the other breast was fully emptied by hand. Breast milk composition is known to dynamically change even during the course of a single feed. Therefore, samples (5 mL) were taken from the entire mixed volume of collected milk and stored in glass bottles (pyrex teflon lid glass bottles) at −20 °C until analyzed.

Finally, concentrations of OPN in the breast milk samples were analyzed using the Quantikine^®^ Human Osteopontin ELISA kit (R&D Systems, Minneapolis, MN, USA) according to the manufacturer’s instructions. The results were read and documented in ng/mL.

### 2.3. Statistical Analysis

Statistical analyses were performed using the IBM Statistical Package for the Social Sciences (SPSS) 24 (IBM Corporation, Armonk, NY, USA) and Microsoft Excel 2016 (Microsoft Corporation, Redmond, WN, USA). BeBiS (Nutrition Information System) 8.1 software (BeBiS, Istanbul, Turkey) was used to analyze the recorded frequencies and consumption quantities of each food group and to calculate the average daily intake of energy and macro- and micronutrients of each subject. The calculated individual energy, macro- and micronutrient levels prior to lactation were transferred to IBM SPSS software (IBM Corporation, Armonk, NY, USA) for statistical analysis.

The conformity of variables to normal distribution was examined using the Kolmogorov–Smirnov test; values of descriptive analyses were expressed as mean ± standard deviation for normally distributed variables and as median and minimum-maximum for abnormally distributed variables. Nominal variables were presented as frequency and percentages.

According to the principles of parametric test assumptions and depending on the statistical characteristics of the digital data analyzed, pairwise comparisons were conducted using the appropriate parametric (independent samples t-test) and non-parametric (Mann–Whitney U test) tests. Similarly, multiple group comparisons of numerical data were performed according to the statistical assumptions of normal distribution, using the appropriate parametric or non-parametric test (ANOVA or Kruskal–Wallis H test, respectively). The chi-square test was used to determine the relationships between categorical variables [30,31].

Correlation tests were performed using Pearson’s correlation when parametric conditions were met, and with the Spearman correlation when the data were non-parametric [30,31]. Linear regression modelling was used to identify factors that may affect levels of OPN in breast milk. Model fit was examined using the necessary compliance statistics. Logarithmic conversion was performed for non-parametric data. In all statistical tests, results were interpreted as statistically significant where p was less than 0.05 [30,31].

## 3. Results

### 3.1. Study Population

The general characteristics of the study population are presented in Table 2. The average age of the participants was 30.2 ± 6.0 years. The participants were evenly divided into five-year age groups. The majority (61.2%) had given birth by the natural cervical vaginal route, while 38.8% underwent Cesarean section for various reasons. Forty per cent of participants stated that they had smoked before pregnancy, compared with 16.5% and 15.3% during pregnancy and lactation, respectively. No subjects reported having used alcohol at any time, neither before pregnancy, nor during pregnancy, nor during lactation.

All study subjects were married or living with a partner during pregnancy and at the time of study participation. The women’s education status was assessed, revealing that 50.6% had been educated to at least high school level. The age distribution of the subjects showed that most experienced their first pregnancy between the ages of 20 and 24 years (48.2%). The median total number of pregnancies was 2.0 (1.0–5.0) and none of the women had experienced repeated miscarriages or any diagnosis of genetic or chronic disease. Including the prevenient gravidity, 64.7% had experienced pregnancy once or twice in total. Mean age at first pregnancy was 23.7 ± 5.2 years. The median number of living children was 2.0 (1.0–4.0) and it was observed that 38.8% of individuals had just borne their first child (Supplementary Tables S1 and S2).

Mean BW was 62.2 ± 8.5 kg at pregnancy onset and 76.5 ± 9.2 kg during pregnancy. During lactation (at the study visit), a mean BW of 67.9 ± 9.6 kg was determined. In the pre-pregnancy period, it was determined that, according to WHO classifications, 60% of subjects had a healthy body weight (18.5–24.9 kg/m^2^), while 5.9% were underweight (<18.5 kg/m^2^), 24.7% were overweight (25.0–29.9 kg/m^2)^ and 9.4% had clinical obesity (≥30 kg/m^2^). Of those subjects whose BMI was normal prior to pregnancy, 25.5% showed an adequate increase in BW during pregnancy, whereas weight gain was insufficient in 33.3% and excessive in 41.2%. Inadequate BW gain was not observed in any subjects classified as having overweight or obesity at pregnancy onset, while their rate of excess weight gain was increased compared with individuals whose BMI was within the healthy range (71.4% and 62.5%, respectively). Of participants classified as underweight according to the pre-pregnancy BMI classification, 60% showed insufficient weight gain during pregnancy. In the lactation period (study visit, 3rd month of lactation), whereas none of the mothers were found to be underweight, the proportion of individuals with a healthy BW decreased (45.9%) and a larger proportion were found to have obesity (17.5%) or overweight (45.6%) (Table 2).

### 3.2. Maternal Energy and Macronutrient Intake

The individual mean energy intake levels (based on the FFQ as described above) ranged from 1874.8 kcal/day to 4058.2 kcal/day. The overall mean level for the cohort was 2951 ± 541.4 kcal/day. Mean individual daily protein intake ranged from 49.4 ± 145.4 g to 98.1 ± 20.4 g. In mean, 45.4 ± 10.0 g of daily protein intake comprised vegetable or plant proteins, while total proteins contributed 13.7 ± 2.2% of individual daily energy intake. Daily carbohydrate intake ranged between 209.4 and 569.0 g, with a mean of 354.2 ± 81.2 g. In mean, energy from carbohydrate intake comprised 49.1 ± 5.9% of the daily energy intake of the subjects. The participants’ daily fat intake ranged from 70.1 ± 185.5 g to 123.1 ± 28.6 g, with fat intake constituting 37.2 ± 5.2% of daily energy intake, in mean. The daily energy and macronutrient intake levels of the study participants are presented in Table 3.

### 3.3. Osteopontin Levels in Relation to Maternal Factors

OPN levels measured in the breast milk samples are shown in Table 2. The mean level of OPN in the mothers’ breast milk was determined to be 137.1 ± 56.8 mg/L. No association of breast milk OPN levels was found with maternal age, age at first pregnancy, number of pregnancies, or number of living children (*p* > 0.05). On the other hand, mean breast milk OPN levels were significantly higher (160.6 ± 48.8 mg/L) in mothers who had given birth via the cervical vaginal route compared with those who had borne their babies by Cesarean section (99.9 ± 48.5 mg/L) (*p* < 0.001), independent of the women’s age (Table 4).

No correlation was found between pre-pregnancy BMI classification and breast milk OPN levels. However, it was observed that breast milk OPN concentrations were associated with BMI during lactation, being reduced in mothers with a higher BMI index compared to those with a normal BMI during the lactation period. The mean breast milk OPN levels of mothers with a normal BMI, overweight and obesity during lactation were 156.4 ± 46.2 mg/L, 140.8 ± 61.2 mg/L and 78.9 ± 28.8 mg/L (*p* < 0.001), respectively. A binary sub-group comparison showed the difference in breast milk OPN levels in normal versus overweight mothers in this cohort to be statistically insignificant (*p* > 0.05). In contrast, subjects with obesity were found to have significantly lower concentrations of OPN in their breast milk compared not only with mothers of normal weight, but also with mothers whose lactation BMI was classified as overweight (*p* < 0.05) (Table 2).

**Figure 1:**
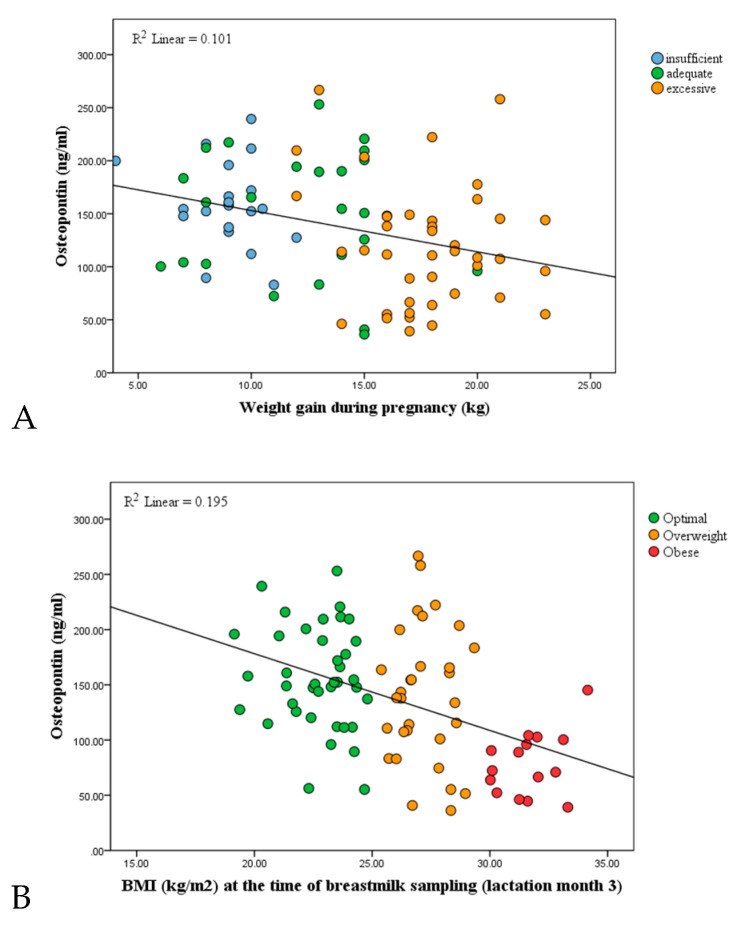
(**A**) Correlation between weight gain during pregnancy and OPN levels in the breast milk of study participants (r = −0.441, *p* < 0.05, Spearman correlation). (**B**) Correlation between BMI during lactation and the mothers’ breast milk OPN levels (r = −0.352, *p* < 0.05, Pearson correlation).

Breast milk OPN levels were additionally analyzed regarding weight gain during pregnancy. OPN levels in study subjects categorized as having gained insufficient weight during pregnancy were 158.2 ± 40.3 mg/L, compared with 149 ± 60.4 mg/L in mothers with adequate weight gain, and 119. 8 ± 57.4 mg/L in those with excessive weight gain. The difference between the groups was found to be statistically significant (*p* < 0.05). In the bivariate group analysis, the difference in breast milk OPN levels between mothers with insufficient weight gain and those with adequate weight gain during pregnancy was not statistically significant. However, mean OPN levels in the group with excessive weight gain were significantly lower than in both the adequate weight gain and the insufficient weight gain groups (*p* < 0.05) (Table 2). Both weight gain during pregnancy and increased BMI during lactation were identified as factors related to a reduction in breast milk OPN concentrations (*p* < 0.05). The relationships of weight gain during pregnancy (A) and lactation BMI (B) to breast milk OPN levels are presented as correlation graphs in Figure 1.

Breast milk OPN concentrations were observed to be significantly lower in mothers who smoked. The mean breast milk OPN levels of individuals who smoked in the pre-pregnancy period were 102.0 ± 41.4 mg/L, a significant reduction compared with the group of mothers who did not smoke 160.4 ± 53.8 mg/L (*p* < 0.05). Similarly, mean levels of breast milk OPN were significantly decreased in mothers who smoked during pregnancy or lactation (103.1 ± 50.6 mg/L and 98.3 ± 49.2 mg/L, respectively) compared with non-smoking mothers (143.8 ± 55.9 mg/L and 144.1 ± 55.5 mg/L, respectively) (Table 2).

### 3.4. Osteopontin Levels in Relation to Maternal Energy and Macronutrient Intake

The relationship between the women’s OPN levels and their average daily energy, macronutrient, and fiber intake during lactation are shown in Table 3. A moderate negative correlation was determined between daily energy intake and breast milk OPN levels (r = −0.406, *p* = < 0.001). In addition, a low-level negative correlation between dietary fat intake and breast milk OPN levels (r = −0.255, *p* = 0.019) was identified. Whereas no significant correlation was observed between breast milk OPN concentrations and either percentage of energy from fat in the diet, or intake of monounsaturated or saturated fatty acids, respectively, a low-level negative correlation between dietary intake of polyunsaturated fatty acids and concentrations of breast milk OPN (r = −0.268, *p* = 0.013) was identified. In addition, while no relationship was found between omega-3 fatty acids and breast milk OPN levels, a moderate correlation was determined between the intake of omega-6 fatty acids during lactation and the mothers’ breast milk OPN levels (r = −0.301, *p* = 0.005). A negative moderate correlation was found between daily carbohydrate intake and breast milk OPN levels (r = −0.333, *p* = 0.002). While no relationship was found between OPN concentrations in breast milk and the percentage of energy from carbohydrate intake in the diet, a negative low-level correlation was determined between OPN levels and the intake of dietary sucrose (r = −0.262, *p* = 0.015). Total dietary fiber intake and breast milk OPN levels showed no correlation. However, when soluble and insoluble fibers were examined separately, a negative correlation was found between OPN concentrations and soluble fiber intake (r = −0.292, *p* = 0.007).

### 3.5. Osteopontin Levels in Relation to Anthropometric Measurements at 0–3 Months and Number of Hospital Admissions Related to Febrile Illness of the Infant

Correlations between the mothers’ OPN levels and their baby’s anthropometric measurements at birth, and at the first and third months, and the number of hospital admissions of those babies due to febrile illness from birth to the third month of life are shown in Table 5. Significant correlations were determined between BW, length, and head circumference, respectively, and breast milk OPN levels at the first (r = 0.442, *p* = < 0.001; r = −0.284, *p* = < 0.001 and r = −0.392, *p* = < 0.001) and third months (r = 0.501, *p* = < 0.001; r = −0.450, *p* = < 0.001 and r = −0.498, *p* = < 0.001) of lactation. No statistically significant relationship was defined between anthropometric measurements of infants at birth and breast milk OPN levels. In addition, a moderate negative correlation between the number of hospital admissions due to febrile illness from birth to 3 months and breast milk concentrations of OPN (r = −0.599, *p* < 0.001) was identified.

## 4. Discussion

The aims of this research were to measure OPN levels in mature breast milk, to investigate which maternal factors may influence these levels, and to identify possible relationships between breast milk OPN concentrations and neonate health. Although the bioactive components of breast milk have been a subject of discussion for many years, the functions of OPN and its relation to maternal and infant health have yet to be completely elucidated. OPN in breast milk was first described by Senger et al. in 1989 [32] and Sorensen et al. showed the presence of OPN in cow’s milk in 1993 [33]. Shortly afterwards, in 2004, Nagatomo et al. detected human breast milk OPN concentrations of 1493.4 mg/L at 3–7 days postpartum and 896.3 mg/L after one month [13], results that would indicate that OPN comprises approximately 10% of human milk protein. In 2009, however, Schack et al. questioned these findings and performed a new study showing an OPN concentration of 138 mg/L in breast milk and reporting that it made up 1–3% of breast milk protein (ca. 100–300 mg/L) [13]. In 2018, Bruun et al. [34] found that the median concentration of OPN contained in 829 milk samples taken from 629 mothers from different countries was 157.0 mg/L. In the present study, the mean concentration of OPN was determined to be 137.1 ± 56.8 mg/L. This value is consistent with previous studies of human milk. and particularly close to the levels reported by Schack et al.

To date, only a few studies have focused on the functions of human breast milk OPN, and to our knowledge, this is the first to investigate the relationship between OPN levels in breast milk and maternal factors. In our study sample, breast milk OPN levels were not associated with maternal age, age at first pregnancy, total number of pregnancies, or the number of living children. On the other hand, OPN levels were found to be significantly higher in the breast milk of mothers who gave birth via the natural cervical vaginal route (160.6 ± 48.8 mg/L) compared with mothers who delivered by Cesarean section (99.9 ± 48.5 mg/L), independent of age. There are no previous data correlating concentrations of breast milk OPN with the birth route of infants. Interestingly, however, Ge et al. [35] found that in vitro oxytocin exposure promoted gene expression of OPN. Since oxytocin is highly expressed during vaginal birth, this might explain the higher concentrations of OPN associated with vaginal birth compared with Cesarian, where oxytocin is lacking. Nissen et al. [36] found that after vaginal delivery, mothers showed a more pulsatile oxytocin re-lease pattern during breastfeeding two days after delivery (as well as a marked increase in prolactin levels) compared with women who delivered by Cesarian. Reports suggest that delivery method may also affect other aspects of breast milk composition. Dizdar et al. [37] determined a higher protein content in the colostrum of mothers who had a cervical vaginal delivery compared with mothers who underwent Cesarean section. Hahn et al. [38] reported a significant increase in fat content from 0–3 months in the breast milk of mothers who had undergone Cesarean section. In contrast, a significant increase in breast milk carbohydrate content was found in mothers who gave birth by the cervical vaginal route. A study by Affolter et al. [38] suggested that breast milk immune factors may be affected by Cesarean delivery. Taken together, these studies indicate that delivery route may influence breast milk composition, albeit to a limited extent. Our results are in line with this, suggesting that breast milk OPN levels are related to the birth route. However, since our samples were collected only at a single timepoint, during the 3rd month of lactation, it is possible that they may represent a coincidental finding. Further studies are needed with regular sample collection from a broader and more homogeneous study population, starting with samples of colostrum.

Our data also indicated that during pre-pregnancy, pregnancy and lactation, breast milk OPN levels were significantly lower in the milk of mothers who smoked (*p* < 0.05). However, when interpreting these data, it must be kept in mind that the smokers in all three periods were the same women, some of whom refrained from smoking during pregnancy and lactation. Thus, while these data show an association of smoking with breast milk OPN levels, conclusions cannot be drawn specifically for any one time period. Studies have shown that in lactating mothers who smoke, vitamin C levels are decreased [39], the lipid composition of breast milk is altered, and total fat content is reduced [40]. Mitnerowicz et al. [41] found smoking to have no effect on albumin or lactoferrin levels, but to reduce the volume of breast milk produced. In another study by Bachour et al. [42], smoking did not affect concentrations of albumin, IgA, lactoferrin or casein in breast milk. Uniquely, however, Bachour et al. showed that total breast milk protein levels decreased significantly due to smoking [42]. Our study suggests that the relationship between smoking and breast milk OPN levels is worthy of further investigation.

In the present study cohort, no relationship was determined between pre-pregnancy BMI and levels of breast milk OPN. There are no studies in the literature that directly correlate breast milk OPN with pre-pregnancy BMI. However, Saha et al. [43] reported lactoferrin and lysozyme levels to be similar in breast milk samples taken from women with a BMI below and above 20 kg/m^2^ prior to pregnancy. In another study, pre-pregnancy BMI did not affect breast milk IgA levels [42]. Thus, the lack of correlation between OPN and pre-pregnancy BMI is not an unexpected finding.

Weight gain during pregnancy and BMI during lactation were both found to be associated with breast milk OPN concentrations: These were significantly lower in the milk of mothers who gained excessive weight during pregnancy compared with mothers whose weight gain was in line with or below recommended levels. On the other hand, breast milk OPN levels of mothers with inadequate weight gain and those with healthy weight gain were found to be similar. Similarly, whereas breast milk OPN levels did not differ between the “normal” vs. “overweight” BMI groups during lactation, obesity was significantly associated with reduced OPN concentrations compared with the other two groups. In the literature, we found no study directly investigating breast milk OPN levels and maternal BMI and weight gain during pregnancy. In a study by Yangz et al. [44] focusing on factors influencing lactoferrin levels in breast milk, no relation between maternal BMI and breast milk lactoferrin concentrations was found. While Nayak et al. [45] found no correlation between maternal BMI and breast milk composition, Kugananthan et al. [46] linked increased maternal fat tissue volume to increased concentrations of both protein and hormones in breast milk. Moreover, Whitaker et al. [47] reported enhanced levels of inflammatory markers in the breast milk of women with excessive weight gain during pregnancy. Existing data are thus inconclusive concerning possible links of maternal BMI and weight gain during pregnancy to macro- or micronutrients in the breast milk. Therefore, our findings linking these factors with reduced breast milk OPN levels might simply reflect the fact that the interrelationship between OPN, obesity and inflammation is complex and worthy of further investigation.

Low or moderate diversity of the maternal diet has not, in general, been found to cause major variations in breast milk composition or volume [48,49,50]. Nevertheless, studies have shown that maternal energy intake affects breast milk volume, that maternal fat intake does not influence the composition of fat in breast milk, and that deficiencies of some vitamins and minerals influence vitamin and mineral levels in breast milk [3,45]. Therefore, major differences in maternal nutrition may affect the composition of breast milk. In this study, we sought to examine possible effects of maternal intake of energy and macro- and micronutrients on breast milk OPN levels for the first time. A few studies have indicated that IgA, IgG and lysozyme levels are significantly decreased in the breast milk of mothers with malnutrition compared with mothers of a healthy nutritional status [51,52]. However, the influence of maternal nutrition on immunological properties of breast milk remains unclear. Our data showed low to moderate associations of breast milk OPN concentrations with maternal intake levels of energy, vegetable/plant proteins, total fat, poly-unsaturated fatty acids, carbohydrate, and fiber. Additional studies are required to clarify these relationships further.

Breast milk OPN has been linked to a healthy pattern of growth and development in infants. In rhesus monkeys, Donovan et al. [22] found the growth pattern and bone miner-al density of baby monkeys fed OPN-supplemented formula to be similar to those of exclusively breastfed monkeys. Later, Lönnerdal et al. [24] reported similar findings in a human cohort of 240 neonates. To our knowledge, ours is the first study since to focus on growth patterns of neonates in relation to breast milk OPN levels, albeit without a control group of infants receiving formula feed. Our results are consistent with the existing literature. Generally, infants of mothers with higher breast milk OPN levels had a higher mean BW and length at both one and three months. We found no significant correlation between breast milk OPN levels and the BW and length measurements of infants at birth. However, significant positive correlations between breast milk OPN levels and BW and length were found after one and three months, suggesting that OPN levels in milk may be related to the early growth pattern of neonates. Recent studies also concluded that OPN in human milk could play an important role in brain development and behavior in infancy [53,54], possibly by promoting myelination [54].

The most important function of breast milk OPN in infant health is thought to be its role in regulating immune system development [17]. OPN-producing epithelial cells and macrophages have been found in the actively lactating mammary gland, implicating that high expression of OPN in human milk cells may play an essential role in the immuno-logical development of breastfed infants [26,55]. Both circulating T cells and levels of pro-inflammatory cytokines were found to be reduced in infants fed OPN-supplemented formula compared with infants fed standard formula feed [24,56]. Thus, OPN ap-pears to affect both innate and adaptive immunity. Donovan et al. [22]. reported that pathways of cell proliferation and cell–cell adhesion were upregulated in infants who were breastfed or fed OPN-supplemented formula in comparison to those fed standard formula, thus promoting the production and maturation of immune cells. Plasma OPN is known to play an important role in the immune system response to microbial infections [57]. Mice with OPN deficiency were found to be more susceptible to pathogenic microorganisms such as *Listeria monocytegenes*, *Plasmodium chabuad*, and *Mycobacterium bous* [58,59,60], while in another OPN-deficient murine model, the spontaneous development of colitis was reported [61]. Lönnerdal et al. observed that infants fed OPN-enriched formula had a lower incidence of fever compared with infants fed standard formula [24]. In line with this, we found a significantly reduced incidence of hospital admission due to febrile illness during the first three months of life in the infants of mothers with higher breast milk OPN levels. These data are consistent with the literature and not surprising, given the known immune system-related functions of OPN.

## 5. Conclusions

In conclusion, we found that OPN concentrations in breast milk seem to be affected by maternal factors including dietary intake (especially energy and fiber intake), BMI and smoking. Our data also indicated an influence of the birth route, with higher breast milk OPN levels found in mothers who delivered via cervical vaginal birth compared with Cesarean section. In addition, we found correlations between OPN levels and measures of infant growth in the first three months of life. Overall, our results are in line with previous findings suggesting that OPN might have important effects on infant growth and health. Our data showing reduced postnatal fever-related hospital admissions in babies fed breast milk with higher levels of OPN substantiate a possible role of OPN-associated mechanisms in immune system development in the neonate. In order to gain more clarity on functions of OPN as a bioactive ingredient of human breast milk, additional studies are needed in a broader and more homogeneous study population with sample collection on a regular basis and over a longer time period. Depending on future findings, the concept of supplementing infant formulas with OPN should be further pursued. In the light of our findings, we believe this may indeed prove a viable option and an important contribution to the health and immune system development of infants who, for whatever reason, cannot receive breast milk.

## Figures and Tables

**Table 1:** Recommendations for weight gain during pregnancy according to BMI classification before pregnancy (IOM).

BMI before Pregnancy, kg/m (WHO Classification)		Recommended Weight Gain (kg)
Underweight	<18.5	13–18
Optimal	18.5–24.9	11–16
Overweight	25.0–29.9	7–11
Obese	≥30.0	5–9

**Table 2:** General characteristics of the mothers and corresponding breast milk OPN (Osteopontin) levels.

General Characteristics	n (%)	OPN, mg/L (Mean ± SD)	*p*_1_	*p*_2_
***n***	85 (100.0)	137.1 ± 56.8		
**Age groups**				
19–24 years	20 (23.5)	137.8 ± 65.8	-	0.334
25–29 years	22 (25.9)	141.4 ± 53.8		
30–34 years	17 (20.0)	153.6 ± 49.3		
35–39 years	26 (30.6)	121.9 ± 55.9		
*Age*, *mean± sd*	*30.2 ± 6.0*			
**Birth method**				
Cervical vaginal route	52 (61.2)	160.6 ± 48.8	<0.001 **	-
Cesarean section	33 (38.8)	99.9 ± 48.5		
**BMI (kg/m^2^) classification**				
Pre-pregnancy				
<18.5 (underweight)	5 (5.9)	140.6 ± 37.9	-	0.108
18.5–24.9 (optimal)	51 (60.0)	143.6 ± 53.6		
25.0–29.9 (overweight)	21 (24.7)	111.9 ± 56.8		
≥30 (obese)	8 (9.4)	159.1 ± 73.5		
*BMI, mean* *± sd*	*23.6 ± 3.3*			
Postpartum/Lactation (3^rd^ month)				
<18.5 (underweight)	-	-	-	<0.001 **
18.5–24.9 (optimal)	39 (45.9)	156.4 ± 46.2 ^a^		
25.0–29.9 (overweight)	31 (36.5)	140.8 ± 61.2 ^a^		
≥30 (obese)	15 (17.6)	78.9 ± 28.8 ^b^		
*BMI*, *mean± sd*	*25.8 ± 3.8*			
**Weight gain during pregnancy**				
Insufficient	20 (23.5)	158.2 ± 40.3 ^a^	-	0.020 *
Adequate	24 (28.)	149.0 ± 60.4 ^a^		
Excessive	41 (48.2)	119.8 ± 57.4 ^b^		
**Smoking status**				
Pre-pregnancy				
Yes	34 (40.0)	102.0 ± 41.4	<0.001 **	-
No	51 (60.0)	160.4 ± 53.8		
Pregnancy				
Yes	14 (16.5)	103.1 ± 50.6	0.013 *	-
No	71 (83.5)	143.8 ± 55.9		
Postpartum/Lactation				
Yes	13 (15.3)	98.3 ± 49.2	0.007 *	-
No	72 (84.7)	144.1 ± 55.5		

*p*_1_; independent samples t-test, *p* _2_; ANOVA test, * *p* < 0.05, ** *p* < 0.001. ^a,b^; A statistically significant difference between binary groups is expressed by different lower-case letters (*p*; independent samples *t*-test).

**Table 3:** Maternal energy and macronutrient intake in lactation period (0–3 months) and their correlation with breast milk OPN (Osteopontin) levels at month 3 study visit.

Energy and Macronutrients	OPN (mg/L)			
	Mean ± SD	r	*p*_1_	*p* _2_
**Energy (kcal)**	2951.0 ± 541.4	−0.406	<0.001 **	-
**Protein (g)**	98.1 ± 20.4	−0.154	0.159	-
Protein (% of total energy)	13.7 ± 2.2	0.204	-	0.062
Vegetable/plant protein (g)	45.4 ± 10.0	−0.272	0.012 *	-
**Fat (g)**	123.1 ± 28.6	−0.255	0.019 *	-
Fat (% of total energy)	37.2 ± 5.2	0.013	-	0.907
Saturated fat (g)	41.3 ± 12.2	−0.158	0.148	-
Saturated fat (% of total energy)	12.5 ± 2.6	0.111	-	0.314
MUFAs (g)	49.4 ± 13.8	−0.140	0.201	-
MUFAs (% of total energy)	15.2 ± 3.6	0.123	-	0.262
PUFAs (g)	23.4 ± 9.3	−0.268	0.013 *	-
PUFAs (% of total energy)	7.1 ± 2.4	−0.155	-	0.156
Omega−3 fatty acids (g)	2.3 ± 1.2	−0.143	-	0.191
Omega-6 fatty acids (g)	20.8 ± 8.2	−0.301	-	<0.001 **
Cholesterol (mg)	426.1 ± 167.3	−0.011	-	0.923
**Carbohydrates (g)**	354.2 ± 81.8	−0.338	<0.001 **	-
Carbohydrates (% of total energy)	49.1 ± 5.9	−0.101	0.356 *	-
**Fiber (g)**	32.2 ± 8.1	−0.262	0.015	-
Soluble fiber (g)	10.3 ± 3.0	−0.187	0.087	-
Insoluble fiber (g)	21.4 ± 5.7	−0.292	<0.001 **	-

*p* _1_; Pearson correlation test, *p* _2_ Spearman correlation test, * *p* < 0.05, ** *p* < 0.001, MUFA; Monounsaturated fatty acid, PUFA; Polyunsaturated fatty acid.

**Table 4:** Mean breast milk osteopontin levels according to age and birth method.

Maternal Characteristic		n	Osteopontin Level (mg/L)				*p* _1_
			Mean	Sd	Min	Max	
**Age (Years)**	**Birth Method (Last Pregnancy)**						
19–24	Cervical vaginal route	13	163.7	55.0	83.0	266.7	<0.001 **
	Caesarean section	7	76.6	30.4	39.2	114.8	
25–29	Cervical vaginal route	14	147.3	45.5	83.8	215.9	0.044 *
	Caesarean section	8	121.1	68.2	36.2	258.0	
30–34	Cervical vaginal route	11	172.5	49.7	72.3	239.3	0.010 *
	Caesarean section	6	119.0	24.7	90.5	148.2	
35–39	Cervical vaginal route	14	155.1	46.0	74.6	222.3	<0.001 **
	Caesarean section	12	83.1	39.3	40.8	149.0	

*p* _1_; independent samples *t*-test, * *p* < 0.05, ** *p* < 0.001.

**Table 5:** Anthropometric measurements of infants at birth, and at the 1st and 3rd months, the number of hospital admissions of the baby due to febrile illness from birth to 3rd month of life and their correlations to breast milk OPN (Osteopontin) levels.

Characteristics of the Infants	OPN (mg/L)			
	Mean ± SD	r	*p* _1_	*p* _2_
**Body weight (g)**				
At birth	3266.2 ± 351.1	−0.70	-	0.523
1st month	4452.9 ± 450.0	0.442	<0.001 **	-
3rd month	6297.9 ± 499.5	0.501	<0.001 **	-
**Length (cm)**				
At birth	49.7 ± 1.9	0.185	-	0.090
1st month	54.5 ±1.5	0.284	<0.001 **	-
3rd month	62.8 ± 2.9	0.450	<0.001 **	-
**Head circumference (cm)**				
At birth	34.4 ± 0.9	0.148	-	0.177
1st month	37.1 ± 1.1	0.392	<0.001 **	-
3rd month	41.0 ± 1.1	0.498	<0.001 **	-
**Hospital admissions due to fever (n), 0–3 months**	1.0 ± 1.7	−0.599		<0.001 **

*p* _1_; Pearson correlation test, *p* _2_ Spearman correlation test, * *p* < 0.05, ** *p* < 0.001.

## Data Availability

The data presented in this study are available on request from the corresponding author. The data are not publicly available due to data protection regulations.

## Supplementary Materials

The following are available online at https://www.mdpi.com/article/10.3390/nu13082670/s1: Table S1: Distribution of mothers according to general socioeconomic characteristics. Table S2: Mean breast milk osteopontin levels according to specific maternal characteristics.
