# Supplementary material for: Osteopontin Levels in Human Milk Are Related to Maternal Nutrition and Infant Health and Growth
Source: Nutrients. 2021 Jul 31;13(8):2670. doi: 10.3390/nu13082670 (PMC8402120; doi:10.3390/nu13082670)
Supplement: Supplementary file 1 [file nutrients-13-02670-s001.zip › nutrients-1308262-supplementary.pdf]

Table S1. Distribution of mothers according to general socioeconomic characteristics

General Socioeconomic Characteristics	Mothers (n = 85)	
	Number	%
**Educational Status**		
Primary education	15	17.6
Secondary education	27	31.8
High school education	33	38.8
College/Univ. graduate	10	11.8
**Marital Status**		
Married	85	100.0
Single	0	0
**Employment Status**		
Employed	23	27.1
Housewife	62	72.9
**Age at first pregnancy (years)**		
15–19	18	21.2
20–24	41	48.2
25–29	15	17.6
30–34	5	5.9
35–39	6	7.1
**$\bar{X}$ ± SD (years)**	**23.7 ± 5.2**	
**Total Number of Pregnancies**		
1–2	55	64.7
3–4	24	28.2
≥5	6	7.1
**Median (min.–max.)**	**2.0 (1.0–7.0)**	
**Number of Living Children**		
1 child	33	38.8
2 children	30	35.3
3 children	19	22.4
4 children	3	3.5
**Median (min.–max.)**	**2.0 (1.0–4.0)**	

Table S2. Mean breast milk osteopontin levels according to specific maternal characteristics

Maternal Characteristic	n	Osteopontin Level (mg/L)				p_1	p_2
		mean	SD	Min	Max		
**Age (years)**							
19–24	20	137.8	65.8	39.2	266.7	-	0.334
25–29	22	141.4	53.8	36.2	258.0		
30–34	17	153.6	49.3	72.3	239.3		
35–39	26	121.9	55.9	40.8	222.3		
**Birth method (last pregnancy)**							
Natural cervical vaginal route	52	160.6	48.8	72.3	266.7	<0.001 **	
Caesarean section	33	99.9	48.5	36.2	258.0		
**Age at first pregnancy (years)**							
15–19	18	130.5	39.3	55.3	209.5	-	0.948
20–24	41	140.2	64.3	36.2	266.7		
25–29	15	141.3	53.6	56.3	215.9		
30–34	5	137.6	63.4	44.7	222.3		
35–39	6	124.2	64.4	40.8	203.7		
**Total Number of Pregnancies**							
1–2	55	139.3	58.6	36.2	266.7	0.678	-
3–4	24	129.1	52.6	46.2	222.3		
5 and more	6	148.5	61.9	52.2	239.3		
**Number of Living Children**							
1 child	33	132.6	62.3	36.2	266.7	-	0.541
2 children	30	141.3	54.5	44.7	258.0		
3 children	19	144.4	54.0	51.5	239.3		
4 children	3	96.9	22.2	72.3	115.5		

p_1; independent samples *t*-test, p_2; ANOVA test, * $p < 0.05$, ** $p < 0.001$.
